# Tryptophan-Containing Cyclic Decapeptides with Activity against Plant Pathogenic Bacteria

**DOI:** 10.3390/molecules22111817

**Published:** 2017-10-26

**Authors:** Cristina Camó, Maria Torné, Emili Besalú, Cristina Rosés, Anna D. Cirac, Gemma Moiset, Esther Badosa, Eduard Bardají, Emilio Montesinos, Marta Planas, Lidia Feliu

**Affiliations:** 1 LIPPSO, Departament de Química, University of Girona, Maria Aurèlia Capmany 69, 17003 Girona, Spain; cristina.camo@udg.edu (C.C.); mariiatorne@gmail.com (M.T.); c.roses.subiros@gmail.com (C.R.); anna.dcirac@gmail.com (A.D.C.); gemmamoiset@gmail.com (G.M.); 2 Institut de Química Computacional i Catàlisi i Departament de Química, University of Girona, Maria Aurèlia Capmany 69, 17003 Girona, Spain; emili.besalu@udg.edu; 3 Laboratory of Plant Pathology, Institute of Food and Agricultural Technology-CIDSAV-XaRTA, University of Girona, Maria Aurèlia Capmany 61, 17003 Girona, Spain; esther.badosa@udg.edu

**Keywords:** antimicrobial peptides, cyclopeptides, plant pathogens, hemolysis, eukaryotic cytotoxicity, antibacterial activity

## Abstract

A library of 66 cyclic decapeptides incorporating a Trp residue was synthesized on solid phase and screened against the phytopathogenic bacteria *Pseudomonas syringae* pv. *syringae*, *Xanthomonas axonopodis* pv. *vesicatoria*, and *Erwinia amylovora.* The hemolytic activity of these peptides was also evaluated. The results obtained were compared with those of a collection of Phe analogues previously reported. The analysis of the data showed that the presence of the Trp improved the antibacterial activity against these three pathogens. In particular, 40 to 46 Trp analogues displayed lower minimum inhibitory concentration (MIC) values than their corresponding Phe counterparts. Interestingly, 26 Trp-containing sequences exhibited MIC of 0.8 to 3.1 μM against *X. axonopodis* pv. *vesicatoria*, 21 peptides MIC of 1.6 to 6.2 μM against *P. syringae* pv. *syringae* and six peptides MIC of 6.2 to 12.5 μM against *E. amylovora*. Regarding the hemolysis, in general, Trp derivatives displayed a percentage of hemolysis comparable to that of their Phe analogues. Notably, 49 Trp-containing cyclic peptides showed a hemolysis ≤ 20% at 125 μM. The peptides with the best biological activity profile were c(LKKKLWKKLQ) (**BPC086W**) and c(LKKKKWLLKQ) (**BPC108W**), which displayed MIC values ranging from 0.8 to 12.5 μM and a hemolysis ≤ 8% at 125 μM. Therefore, it is evident that these Trp sequences constitute promising candidates for the development of new agents for use in plant protection.

## 1. Introduction

Bacteria are responsible for diseases of plants that produce important economic losses in commercial agriculture industry [1,2]. The treatment of these diseases requires the continued use of chemical pesticides, including copper compounds and antibiotics. However, the main limitations associated to the use of these compounds are their negative effects on the environment and human health and the emergence of resistant bacterial strains [3,4]. The identification of candidates to substitute for or complement current pesticides is a field that has given rise to intensive studies during the last decades.

Antimicrobial peptides are an appealing alternative for the development of new pesticides that meet current health and safety standards, especially due to their broad range of activity and unique mode of action [5,6,7,8,9,10,11,12]. In fact, several linear and cyclic antimicrobial peptides, either natural or de novo designed, have been shown to effectively combat plant pathogens [6,7,13,14,15,16,17,18,19,20,21,22,23]. Cyclic peptides possess advantages over their linear forms mainly related to an increase of the rigidity of the active conformation and of the stability toward protease degradation [23,24,25]. These features are responsible for the more reliable activity of the cyclized molecules.

Combinatorial chemistry constitutes a powerful tool to obtain peptides with improved biological activity against plant pathogens [20,26,27,28,29,30,31,32,33]. In particular, we have employed this methodology to design and synthesize a library of cyclic decapeptides with general structure c(X_5_-Phe-X_3_-Gln) where X is Lys or Leu [20]. Analysis of internal structural features together with a design of experiments (DOE) allowed establishing the sequence pattern for an optimal biological activity, i.e., c(X^1^X^2^X^3^X^4^LysPheLysLysLeuGln) where X^2^ ≠ X^3^ and X^4^ = Lys. Cyclic peptides c(LysLysLeuLysLysPheLysLysLeuGln) (**BPC194**) and c(LysLeuLysLysLysPheLysLysLeuGln) (**BPC198**) were the best derivatives with minimum inhibitory concentration (MIC) values ranging from 3.1 to 25 μM against the plant pathogenic bacteria *Pseudomonas syringae* pv. *syringae*, *Xanthomonas axonopodis* pv. *vesicatoria*, and *Erwinia amylovora*, and low hemolysis (14–17% at 375 μM).

Results obtained from the above library also demonstrated that subtle changes in the peptide sequence influence both the antibacterial and hemolytic activity of this type of antimicrobial peptides [20]. In order to develop analogues with enhanced biological properties, we decided to study the influence of replacing the Phe with a Trp. It is known that Trp plays an important role in the activity of antimicrobial peptides. In fact, previous investigations have reported that Trp-rich antimicrobial peptides have a broad and potent range of biological activity [30,31,34,35,36,37,38,39,40,41,42]. This behavior has been ascribed to the hydrophilic and hydrophobic features of the indole side chain of Trp which facilitate the partition in the bilayer interface through hydrogen bond formation and cation–π interactions. Interestingly, peptides containing Trp residues have been described as potential agents to be used in plant protection. In particular, PAFs are a family of linear Trp-rich hexapeptides that display distinct activity against various fungi including *Penicillium digitatum* and *Magnaporthe oryzae* [26,30,31]. Another example is the hexapeptide **KCM21** active against the bacteria *Pseudomonas syringae* pv. *tomato* DC300 and *Clavibacter michiganensis* subsp. *michiganensis* [40].

Based on the above considerations, herein we designed and synthesized a library of cyclic decapeptides incorporating a Trp residue. This library was screened against the phytopathogenic bacteria *Pseudomonas syringae* pv. *syringae*, *Xanthomonas axonopodis* pv. *vesicatoria* and *Erwinia amylovora.* The hemolytic activity of these peptides was also evaluated.

## 2. Results

In this work, we studied the influence of the replacement of a Phe with a Trp in previously reported antimicrobial cyclic decapeptides [20] to identify sequences with an improved biological activity profile. A 66-member library was prepared and screened for antibacterial and hemolytic activities.

### 2.1. Design and Synthesis

The library of Trp-containing cyclic decapeptides was designed based on our previously described sequences containing a Phe [20]. It included 66 cyclic decapeptides with general structure c(X_5_–Trp–X_3_–Gln) where X is Lys or Leu (Table 1). In particular, 50 peptides resulted from the combination of three Leu and five Lys. The remaining 16 cyclopeptides comprised the substructure Lys^5^TrpLysLysLeuGln^10^ and, at positions 1–4, all possible combinations of Leu and Lys.

The synthesis of this cyclic decapeptide library was performed on solid-phase using a 4-methylbenzhydryl amine (MBHA) resin and following a three-dimensional orthogonal Fmoc/*t*Butyl/Allyl strategy. Lys and Trp residues were incorporated as Fmoc–Lys(Boc)–OH and Fmoc–Trp(Boc)–OH, respectively. Peptide anchoring onto the resin was accomplished via a Fmoc–Glu–OAll residue, which resulted in a Gln after the cleavage step. Peptides were obtained with ~90% purity as determined with high-performance liquid chromatography (HPLC). Peptide identity was confirmed by electrospray ionization mass spectrometry.

### 2.2. Antibacterial and Hemolytic Activities

**Figure 1:**
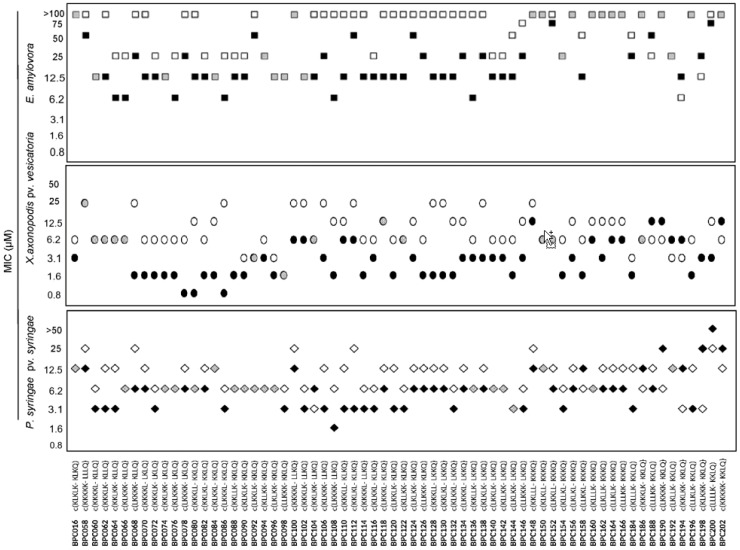
Antibacterial activity of the cyclic peptides incorporating a Trp or a Phe. Sequences and codes of the cyclic peptides are depicted, “-”stands for Trp (W) or Phe (F). Antibacterial activity is given as the minimal concentration that inhibits growth (MIC). The MIC axis is in logarithmic scale and for each sequence the lowest value of the MIC range is represented. Black symbols correspond to peptides with a Trp, white symbols to peptides with a Phe and grey symbols to peptides that exhibit the same MIC values. Data can be found in Table S1 (Supporting Information).

This library was tested for in vitro growth inhibition of *X. axonopodis* pv. *vesicatoria*, *P. syringae* pv. *syringae* and *E. amylovora* (Figure 1). All peptides were active against at least one pathogen with MIC < 25 μM. *X. axonopodis* pv. *vesicatoria* was the most sensitive bacteria to these peptides. Notably, 60 out of 66 sequences displayed MIC < 12.5 μM against this bacterium. Among them, 23 peptides exhibited MIC of 1.6 to 3.1 μM, while **BPC078W**, **BPC080W**, and **BPC086W** were the most active with MIC of 0.8 to 1.6 μM. This library was slightly less active against *P. syringae* pv. *syringae*. However, 52 cyclic peptides showed MIC < 12.5 μM, being **BPC108W** the sequence with the highest activity (MIC of 1.6 to 3.1 μM). Although *E. amylovora* was the least sensitive bacteria to these peptides, 34 of them displayed MIC < 25 μM. Notably, six sequences showed MIC of 6.2 to 12.5 μM.

**Figure 2:**
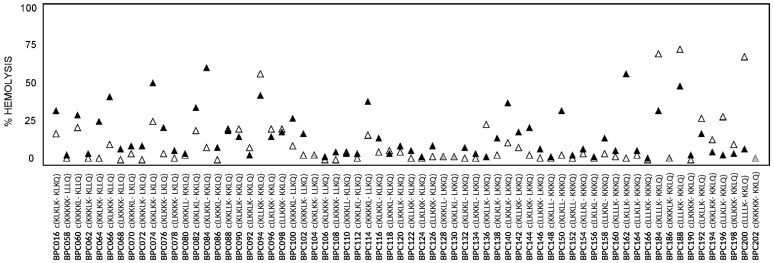
Hemolytic activity of the cyclic peptides incorporating a Trp or a Phe. Sequences and codes of the cyclic peptides are depicted, “-” stands for Trp (W) or Phe (F). Hemolytic activity was measured at 125 μM and is expressed as a percentage compared to melittin as a standard. Black triangles correspond to peptides with a Trp, white triangles to peptides with a Phe and grey triangles to peptides that display the same hemolysis. Data can be found in Table S1 (Supporting Information).

The toxicity of this library was evaluated as ability to lyse erythrocytes in comparison to melittin. Percent hemolysis at 125 μM is included in Figure 2. In general, all cyclic decapeptides were low hemolytic. In particular, 39 out of 66 sequences displayed a hemolysis ≤ 10% at this concentration and ten, hemolysis between 11 and 20%.

Taking all these activity results together, peptides **BPC072W**, **BPC098W**, **BPC120W**, and **BPC132W** showed a good biological activity profile with MIC values ranging from 1.6 to 25 μM and a hemolysis below 20% at 125 μM. The best peptides were **BPC086W** and **BPC108W**, which exhibited MIC values ranging from 0.8 to 12.5 μM. These peptides were also low hemolytic (≤8% at 125 μM).

## 3. Discussion

The identification of synthetic peptides with in vivo activity against economically important plant pathogenic bacteria requires the availability of a large number of sequences highly active in vitro. Cyclic peptides are preferred candidates due to their high conformational rigidity and proteolytic stability [23,24,25]. In a previous work, a library of 66 Phe-containing cyclic decapeptides was synthesized from which sequences with high activity against *E. amylovora*, *P. syringae* pv. *syringae*, and *X. axonopodis* pv. *vesicatoria* were identified, highlighting c(LysLysLeuLysLysPheLysLysLeuGln) (**BPC194**) and c(LysLeuLysLysLysPheLysLysLeuGln) (**BPC198**) (MIC of 3.1 to 25 μM) [20]. With the aim of finding new leads and in view of the key role of Trp in the activity of antimicrobial peptides, we decided to synthesize a library of analogues by replacing the Phe with a Trp.

Several studies have reported that antimicrobial peptides containing a Trp display more potent antimicrobial activity than those with a Phe. In line with this argument, analogues of **LL-37**, cathelicidin-2, cecropin A-melittin hybrid and of temporin 1-Tl with Phe→Trp substitutions exhibited increased antimicrobial activities [37,43,44,45,46]. This trend has been attributed to the bulkier hydrophobic side chain of Trp compared to Phe that would favor the interaction with the cell membrane facilitating a deeper embedding of the peptide [34,35,36,37,38,39,40,41,42].

**Figure 3:**
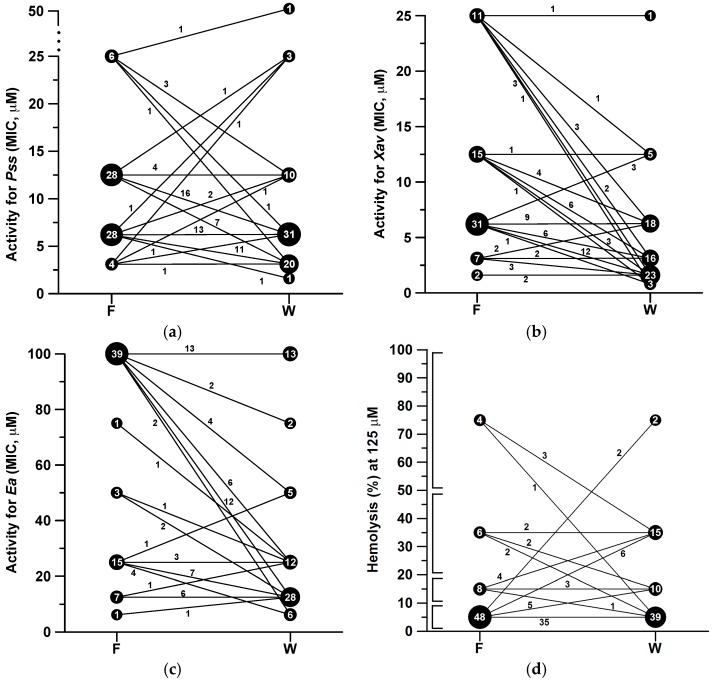
Graphical representation of the antibacterial and hemolytic activity of the cyclic peptides incorporating a Phe or a Trp. Antibacterial activity against (**a**) *P. syringae* pv. *Syringae*; (**b**) *X. axonopodis* pv. *vesicatoria*; (**c**) *E. amylovora*; and (**d**) hemolytic activity. The antibacterial activity is given as the minimal concentration that inhibits growth (MIC), and the lowest value of the MIC range is represented. Hemolytic activity was measured at 125 μM and is expressed as a percentage compared to melittin as a standard. The numbers in the black circles indicate the number of compounds with a given activity, the segment connectors link each Phe-containing peptide with its corresponding Trp-derivative, and the segment labels stand for the number of peptides that follow the same activity trend. The brackets in the y-axis of the hemolytic activity stand for hemolysis percentage ranges.

The analysis of the biological activity of our library of Trp analogues revealed that the presence of the Trp greatly favored the antibacterial activity of this family of cyclic decapeptides against the three phytopathogens tested. In fact, the Trp-containing peptide library included a larger number of active sequences compared to the collection of Phe analogues. In particular, the screening of the Phe-containing cyclic peptide library led to the identification of two sequences with MIC of 1.6 to 3.1 μM against *X. axonopodis* pv. *vesicatoria*, four with MIC of 3.1 to 6.2 μM against *P. syringae* pv. *syringae*, and one with MIC of 6.2 to 12.5 μM against *E. amylovora*. In the case of the Trp analogues, these MIC values were observed for 23, 20 and 6 cyclic peptides, respectively. Accordingly, as shown in Figure 3, when the activity of each Phe-containing peptide (left column) is represented and correlated with that of its Trp derivative (right column), a negative slope was obtained in most cases clearly pointing out to an increase of the activity. This trend was observed for 40, 41 and 46 Trp analogues against *P. syringae* pv. *syringae*, *E. amylovora*, and *X. axonopodis* pv. *vesicatoria*, respectively, which displayed lower MIC values than their corresponding Phe counterparts. Moreover, for 15 to 22 compounds, the Phe→Trp substitution did not influence the activity, as shown from the resulting horizontal lines in Figure 3. Remarkably, within the Trp-containing cyclic peptides we found sequences with highest activity than the Phe derivatives against *X. axonopodis* pv. *vesicatoria* (**BPC078W**, **BPC080W**, and **BPC086W**, MIC of 0.8 to 1.6 μM) and *P. syringae* pv. *syringae* (**BPC108W**, MIC of 1.6 to 3.1 μM).

Regarding the hemolysis, previous studies from the literature revealed that a general activity trend cannot be defined when replacing a Phe by a Trp. On the one hand, Trp has been described to be crucial for the hemolytic activity of peptides such as melittin, mastoparan B, and **EFK17**, which has been attributed to the ability of Trp to assume a defined orientation when binding to cholesterol [43,47,48]. On the other hand, the incorporation of Phe→Trp substitutions in temporin 1-Tl and in a cecropin A-melittin hybrid has led to a small decrease of the hemolytic activity [37,46]. In the present study, it was observed that the presence of a Trp did not significantly influence the hemolytic activity. In fact, as shown in Figure 3, 40 Trp derivatives displayed a percentage of hemolysis comparable to that of their Phe counterparts. Moreover, 49 Trp-containing cyclic peptides vs. 56 Phe analogues showed a hemolysis ≤ 20% at 125 μM.

The results obtained support the notion that subtle changes in a peptide sequence may lead to a significant modification of its biological activity [20,22]. Thus, for example, the Phe→Trp substitution in the lead peptides c(LysLysLeuLysLysPheLysLysLeuGln) (**BPC194**) and c(LysLeuLysLysLysPheLysLysLeuGln) (**BPC198**) caused a reduction of the activity. In contrast, this substitution in c(LeuLysLysLysLeuPheLysLysLeuGln) (**BPC086**) and c(LeuLysLysLysLysPheLeuLeuLysGln) (**BPC108**), which were poorly active, afforded the Trp analogues **BPC086W** and **BPC108W** with the best biological activity profile. These lead peptides exhibited high activity against the three pathogens of this study with MIC values ranging from 0.8 to 12.5 μM and were also low hemolytic (≤8% at 125 μM).

## 4. Materials and Methods

### 4.1. Chemicals and Instruments

The 9-fluorenylmethoxycarbonyl (Fmoc)-protected amino acid derivatives, coupling reagents, 4-methylbenzhyldrylamine (MBHA) resin hydrochloride (0.4 mmol/g), and trifluoroacetic acid (TFA) were obtained from Iris Biotech (Marktredwitz, Germany). Piperidine, Pd(PPh_3_)_4_, sodium *N,N*-diethyldithiocarbamate, triisopropylsilane (TIS), CHCl_3_, *N*-methylmorpholine (NMM), and *N,N*-diisopropylethylamine (DIEA) were from Sigma-Aldrich Corporation (Madrid, Spain). Acetic acid was from Panreac (Castellar del Vallès, Spain). *N*,*N*-Dimethylformamide (DMF), *N*-methyl-2-pyrrolidinone (NMP), and solvents for reverse-phase high-performance liquid chromatography (RP-HPLC) were obtained from Scharlau (Sentmenat, Spain).

HPLC analysis of the peptides was performed using a Dionex liquid chromatography instrument (Dionex, Germering, Germany) with detection at 220 nm. A Kromasil 100 C_18_ (40 mm × 4.6 mm, 3.5 μm, Agilent Technologies, Barcelona, Spain) column was employed and the analysis was carried out with a 2–100% B linear gradient over 17 min at a flow rate of 1 mL/min (solvent A: 0.1% aqueous trifluoroacetic acid (TFA); solvent B: 0.1% TFA in CH_3_CN).

Compounds were characterized by electrospray ionization (ESI) mass spectrometry (MS) analyses using an Esquire 6000 ESI ion Trap LC/MS (Bruker Daltonics, Madrid, Spain) instrument with an electrospray ion source operated in the positive ESI(+) ion mode (Serveis Tècnics de Recerca of the University of Girona, Spain). The instrument was equipped with an HPLC autosampler (Agilent Technologies, Barcelona, Spain) and a 1100 Series HPLC pump (Agilent Technologies, Barcelona, Spain). Samples (5 μL) were introduced into the mass spectrometer ion source directly. The mobile phase consisted of 80:20 CH_3_CN/H_2_O run at a flow rate of 100 μL/min.

Characterization of the compounds by high resolution mass spectrometry (HRMS) analysis (Serveis Tècnics de Recerca of the University of Girona, Spain) was carried out employing a Bruker MicrOTOF-Q IITM instrument (Bruker, Madrid, Spain) with a hybrid quadrupole time-of-flight mass spectrometer and it was operated in the positive ESI(+) ion mode. Samples were introduced into the ion source through direct infusion, being externally calibrated using sodium formate.

### 4.2. Synthesis of the Cyclic Decapeptide Library

The MBHA resin (3.5 g, 0.4 mmol/g) was swollen with CH_2_Cl_2_ (1 × 20 min) and DMF (1 × 20 min), and washed with piperidine/DMF (3:7, 1 × 5 min), DMF (6 × 1 min) and CH_2_Cl_2_ (3 × 1 min). Then, the resin was treated with Fmoc-Rink-amide linker (4 equiv), Oxyma (4 equiv) and DIPCDI (4 equiv) in DMF overnight. After this time, the resin was washed with DMF (6 × 1 min) and CH_2_Cl_2_ (3 × 1 min). After Fmoc removal with piperidine/DMF (3:7, 2 + 10 min) and washes with DMF (6 × 1 min) and CH_2_Cl_2_ (2 × 1 min), Fmoc-Glu-OAll was coupled using the conditions described above. This trifunctional amino acid allows peptide anchoring onto the solid support and results in a Gln residue after peptide cleavage. The elongation of the peptide sequence was performed by sequential Fmoc removal and coupling steps. Once the lineal sequence was completed, the C-terminus allyl group was removed by treatment with Pd(PPh_3_)_4_ in CHCl_3_/AcOH/NMM (3:2:1) for 3 h under nitrogen. Then, the resin was washed with tetrahydrofuran (3 × 2 min), DMF (3 × 2 min), DIEA/CH_2_Cl_2_ (1:19, 3 × 2 min), sodium *N,N*-diethyldithiocarbamate (0.03 M in DMF, 3 × 15 min) and DMF (10 × 1 min). After Fmoc removal with piperidine/NMP (3:7, 1 × 2 min + 2 × 10 min), cyclization was performed by treating the resin with [ethylcyano(hydroxyimino)acetato-O^2^] tri-1-pyrrolidinylphosphonium hexafluorophosphate (PyOxim), Oxyma and DIEA in NMP for 24 h. Following washes with NMP (6 × 1 min) and CH_2_Cl_2_ (2 × 1 min), cyclic peptides were cleaved from the resin by treatment with TFA/phenol/H_2_O/TIS (92.5:2.5:2.5:2.5) for 2 h. The cleavage cocktail was then removed under nitrogen and after diethyl ether extraction, cyclic peptides were dissolved in H_2_O and lyophilized. All the peptides were analyzed by HPLC, and characterized by ESI-MS and HRMS.

### 4.3. Bacterial Strains and Growth Conditions

*Erwinia amylovora* PMV6076 (Institut National de la Recherche Agronomique, Angers, France), *Pseudomonas syringae* pv. *syringae* EPS94 (Institut de Tecnologia Agroalimentària, Universitat de Girona, Spain), and *Xanthomonas axonopodis* pv. *vesicatoria* 2133-2 (Instituto Valenciano de Investigaciones Agrarias, Valencia, Spain) were used as bacterial strains. They were stored and cultivated as previously described [20].

### 4.4. Antibacterial Activity

The minimum inhibitory concentration (MIC) was analyzed as previously described at 0.8, 1.6, 3.1, 6.2, 12.5, 25 and 50 μM [20].

### 4.5. Hemolytic Activity

The hemolytic activity of the compounds was evaluated at 50 and 125 μM by determining hemoglobin release from erythrocyte suspensions of human blood (5% *v*/*v*) (Oxoid) using absorbance at 540 nm, as previously described [20]. Three replicates for each peptide concentration were used.

## 5. Conclusions

This work highlights that the single replacement of a Phe with a Trp in a library of cyclic decapeptides led to analogues with improved antibacterial activity against the economically important phytopathogenic bacteria *P. syringae* pv. *syringae*, *X. axonopodis* pv. *vesicatoria*, and *E. amylovora*. Moreover, this substitution did not result in a significant modification of the hemolysis. Trp-containing cyclic peptides with high activity against these bacteria (MICs from 0.8 to 12.5 μM), and low cytotoxicity (≤8% at 125 μM) were identified. Therefore, these Trp analogues have considerable potential for future development of antimicrobial agents for plant protection.

## Figures and Tables

**Table 1:** Sequences, retention times and purities on high-performance liquid chromatography (HPLC), and mass spectrometry data of cyclic peptides incorporating a Trp.

Peptide	Sequence	*t*_R_ (min) ^a^	Purity (%) ^b^	HRMS
**BPC016W**	c(KLKLKWKLKQ)	6.19	91	647.9420 [M + 2H]^2+^, 1294.8736 [M + H]^+^
**BPC058W**	c(KKKKKWLLLQ)	6.16	>99	647.9400 [M + 2H]^2+^, 1294.8709 [M + H]^+^
**BPC060W**	c(KKKKLWKLLQ)	6.34	>99	647.9389 [M + 2H]^2+^, 1294.8709 [M + H]^+^
**BPC062W**	c(KKKLKWKLLQ)	6.15	>99	647.9371 [M + 2H]^2+^, 1294.8681 [M + H]^+^
**BPC064W**	c(KKLKKWKLLQ)	6.35	>99	647.9427 [M + 2H]^2+^, 1294.8752 [M + H]^+^
**BPC066W**	c(KLKKKWKLLQ)	6.48	88	647.9423 [M + 2H]^2+^, 1294.8752 [M + H]^+^
**BPC068W**	c(LKKKKWKLLQ)	6.00	>99	647.9424 [M + 2H]^2+^, 1294.8700 [M + H]^+^
**BPC070W**	c(KKKKLWLKLQ)	6.04	>99	647.9411 [M + 2H]^2+^, 1294.8716 [M + H]^+^
**BPC072W**	c(KKKLKWLKLQ)	6.04	>99	647.9414 [M + 2H]^2+^, 1294.8718 [M + H]^+^
**BPC074W**	c(KKLKKWLKLQ)	6.33	90	647.9422 [M + 2H]^2+^, 1294.8692 [M + H]^+^
**BPC076W**	c(KLKKKWLKLQ)	6.11	96	647.9423 [M + 2H]^2+^, 1294.8737 [M + H]^+^
**BPC078W**	c(LKKKKWLKLQ)	5.94	>99	647.9410 [M + 2H]^2+^, 1294.8709 [M + H]^+^
**BPC080W**	c(KKKLLWKKLQ)	5.90	>99	647.9396 [M + 2H]^2+^, 1294.8694 [M + H]^+^
**BPC082W**	c(KKLKLWKKLQ)	6.22	88	647.9424 [M + 2H]^2+^, 1294.8727 [M + H]^+^
**BPC084W**	c(KLKKLWKKLQ)	6.42	92	647.9411 [M + 2H]^2+^, 1294.8706 [M + H]^+^
**BPC086W**	c(LKKKLWKKLQ)	5.98	91	647.9405 [M + 2H]^2+^, 1294.8696 [M + H]^+^
**BPC088W**	c(KKLLKWKKLQ)	6.09	88	647.9419 [M + 2H]^2+^, 1294.8700 [M + H]^+^
**BPC090W**	c(KLKLKWKKLQ)	6.03	>99	647.9426 [M + 2H]^2+^, 1294.8740 [M + H]^+^
**BPC092W**	c(LKKLKWKKLQ)	5.89	>99	647.9421 [M + 2H]^2+^, 1294.8706 [M + H]^+^
**BPC094W**	c(KLLKKWKKLQ)	6.39	83	647.9410 [M + 2H]^2+^, 1294.8693 [M + H]^+^
**BPC096W**	c(LKLKKWKKLQ)	6.07	88	647.9418 [M + 2H]^2+^, 1294.8712 [M + H]^+^
**BPC098W**	c(LLKKKWKKLQ)	6.07	>99	647.9418 [M + 2H]^2+^, 1294.8714 [M + H]^+^
**BPC100W**	c(KKKKLWLLKQ)	5.84	>99	647.9411 [M + 2H]^2+^, 1294.8695 [M + H]^+^
**BPC102W**	c(KKKLKWLLKQ)	6.38	96	647.9405 [M + 2H]^2+^, 1294.8694 [M + H]^+^
**BPC104W**	c(KKLKKWLLKQ)	6.28	>99	647.9419 [M + 2H]^2+^, 1294.8700 [M + H]^+^
**BPC106W**	c(KLKKKWLLKQ)	5.89	>99	647.9421 [M + 2H]^2+^, 1294.8640 [M + H]^+^
**BPC108W**	c(LKKKKWLLKQ)	6.02	96	647.9420 [M + 2H]^2+^, 1294.8747 [M + H]^+^
**BPC110W**	c(KKKLLWKLKQ)	6.16	>99	647.9411 [M + 2H]^2+^, 1294.8743 [M + H]^+^
**BPC112W**	c(KKLKLWKLKQ)	6.09	>99	647.9425 [M + 2H]^2+^, 1294.8746 [M + H]^+^
**BPC114W**	c(KLKKLWKLKQ)	6.20	84	647.9423 [M + 2H]^2+^, 1294.8750 [M + H]^+^
**BPC116W**	c(LKKKLWKLKQ)	6.06	89	647.9425 [M + 2H]^2+^, 1294.8730 [M + H]^+^
**BPC118W**	c(KKLLKWKLKQ)	6.10	>99	647.9400 [M + 2H]^2+^, 1294.8707 [M + H]^+^
**BPC120W**	c(LKKLKWKLKQ)	6.11	89	647.9415 [M + 2H]^2+^, 1294.8725 [M + H]^+^
**BPC122W**	c(KLLKKWKLKQ)	6.20	>99	647.9398 [M + 2H]^2+^, 1294.8708 [M + H]^+^
**BPC124W**	c(LKLKKWKLKQ)	5.92	97	647.9414 [M + 2H]^2+^, 1294.8727 [M + H]^+^
**BPC126W**	c(LLKKKWKLKQ)	6.04	87	647.9417 [M + 2H]^2+^, 1294.8737 [M + H]^+^
**BPC128W**	c(KKKLLWLKKQ)	5.92	>99	647.9424 [M + 2H]^2+^, 1294.8764 [M + H]^+^
**BPC130W**	c(KKLKLWLKKQ)	6.01	>99	647.9423 [M + 2H]^2+^, 1294.8745 [M + H]^+^
**BPC132W**	c(KLKKLWLKKQ)	5.93	>99	647.9418 [M + 2H]^2+^, 1294.8753 [M + H]^+^
**BPC134W**	c(LKKKLWLKKQ)	5.87	>99	647.9411 [M + 2H]^2+^, 1294.8731 [M + H]^+^
**BPC136W**	c(KKLLKWLKKQ)	6.87	90	647.9401 [M + 2H]^2+^, 1294.8697 [M + H]^+^
**BPC138W**	c(KLKLKWLKKQ)	5.98	>99	647.9418 [M + 2H]^2+^, 1294.8727 [M + H]^+^
**BPC140W**	c(LKKLKWLKKQ)	6.95	81	647.9406 [M + 2H]^2+^, 1294.8683 [M + H]^+^
**BPC142W**	c(KLLKKWLKKQ)	6.76	87	647.9424 [M + 2H]^2+^, 1294.8755 [M + H]^+^
**BPC144W**	c(LKLKKWLKKQ)	6.72	87	647.9381 [M + 2H]^2+^, 1294.8674 [M + H]^+^
**BPC146W**	c(LLKKKWLKKQ)	6.45	90	647.9419 [M + 2H]^2+^, 1294.8703 [M + H]^+^
**BPC148W**	c(KKLLLWKKKQ)	5.81	97	647.9461 [M + 2H]^2+^, 1294.8842 [M + H]^+^
**BPC150W**	c(KLKLLWKKKQ)	5.92	91	647.9405 [M + 2H]^2+^, 1294.8741 [M + H]^+^
**BPC152W**	c(LKKLLWKKKQ)	5.90	85	647.9411 [M + 2H]^2+^, 1294.8706 [M + H]^+^
**BPC154W**	c(KLLKLWKKKQ)	6.03	87	647.9435 [M + 2H]^2+^, 1294.8759 [M + H]^+^
**BPC156W**	c(LKLKLWKKKQ)	6.37	>99	647.9422 [M + 2H]^2+^, 1294.8723 [M + H]^+^
**BPC158W**	c(LLKKLWKKKQ)	6.12	86	647.9425 [M + 2H]^2+^, 1294.8738 [M + H]^+^
**BPC160W**	c(KLLLKWKKKQ)	5.96	88	647.9425 [M + 2H]^2+^, 1294.8739 [M + H]^+^
**BPC162W**	c(LKLLKWKKKQ)	6.00	93	647.9388 [M + 2H]^2+^, 1294.8701 [M + H]^+^
**BPC164W**	c(LLKLKWKKKQ)	6.38	>99	647.9417 [M + 2H]^2+^, 1294.8723 [M + H]^+^
**BPC166W**	c(LLLKKWKKKQ)	6.38	90	647.9423 [M + 2H]^2+^, 1294.8742 [M + H]^+^
**BPC184W**	c(KLLLKWKKLQ)	6.97	93	640.4371 [M + 2H]^2+^, 1279.8604 [M + H]^+^
**BPC186W**	c(KKKLKWKKLQ)	5.76	>99	655.4473 [M + 2H]^2+^, 1309.8832 [M + H]^+^
**BPC188W**	c(LLLKKWKKLQ)	6.97	93	640.4352 [M + 2H]^2+^, 1279.8585 [M + H]^+^
**BPC190W**	c(LKKKKWKKLQ)	5.70	>99	655.4471 [M + 2H]^2+^, 1309.8811 [M + H]^+^
**BPC192W**	c(LKLLKWKKLQ)	6.30	90	640.4380 [M + 2H]^2+^, 1279.8633 [M + H]^+^
**BPC194W**	c(KKLKKWKKLQ)	6.21	91	655.4456 [M + 2H]^2+^, 1309.8802 [M + H]^+^
**BPC196W**	c(LLKLKWKKLQ)	6.60	99	640.4362 [M + 2H]^2+^, 1279.8610 [M + H]^+^
**BPC198W**	c(KLKKKWKKLQ)	6.26	93	655.4468 [M + 2H]^2+^, 1309.8813 [M + H]^+^
**BPC200W**	c(LLLLKWKKLQ)	7.40	96	632.9259 [M + 2H]^2+^, 1264.8505 [M + H]^+^
**BPC202W**	c(KKKKKWKKLQ)	5.51	>99	662.9523 [M + 2H]^2+^, 1324.8950 [M + H]^+^

^a^ HPLC retention time; ^b^ Percentage determined by HPLC at 220 nm from the crude reaction mixture. HRMS: high resolution mass spectrometry.

## Supplementary Materials

Supplementary Materials are available online. Table S1: Antibacterial activity (MIC) and cytotoxicity of cyclic peptides incorporating a Trp.
