# Supplementary material for: Tryptophan-Containing Cyclic Decapeptides with Activity against Plant Pathogenic Bacteria
Source: Molecules. 2017 Oct 26;22(11):1817. doi: 10.3390/molecules22111817 (PMC6150173; doi:10.3390/molecules22111817)
Supplement: Supplementary file 1 [file molecules-22-01817-s001.pdf]

Supplementary Materials

Tryptophan-Containing Cyclic Decapeptides with Activity against Plant Pathogenic Bacteria

Cristina Camó, Maria Torné, Emili Besalú, Cristina Rosés, Anna D. Cirac, Gemma Moiset, Esther Badosa, Eduard Bardají *, Emilio Montesinos *, Marta Planas *, and Lidia Feliu *

Table of contents

Antibacterial activity and hemolysis of the cyclic peptides incorporating a tryptophan.

Table S1. Antibacterial activity (MIC) and cytotoxicity of cyclic peptides incorporating a Trp.

Peptide		MIC (μM)			Hemolysis (%)[a]	
Code	Sequence	*Pss*[b]	*Xav*[b]	*Ea*[b]	50 μM	125 μM
**BPC016W**	c(KLKLKWKLKQ)	12.5-25	3.1-6.2	>100	25	32
**BPC058W**	c(KKKKKWLLLQ)	12.5-25	25-50	50-75	2	3
**BPC060W**	c(KKKKLWKLLQ)	3.1-6.2	6.2-12.5	12.5-25	22	29
**BPC062W**	c(KKKLKWKLLQ)	3.1-6.2	6.2-12.5	12.5-25	3	4
**BPC064W**	c(KKLKKWKLLQ)	3.1-6.2	6.2-12.5	6.2-12.5	17	25
**BPC066W**	c(KLKKKWKLLQ)	6.2-12.5	6.2-12.5	6.2-12.5	33	41
**BPC068W**	c(LKKKKWKLLQ)	6.2-12.5	1.6-3.1	25-50	6	7
**BPC070W**	c(KKKKLWLKLQ)	6.2-12.5	1.6-3.1	12.5-25	6	9
**BPC072W**	c(KKKLKWLKLQ)	3.1-6.2	1.6-3.1	12.5-25	7	9
**BPC074W**	c(KKLKKWLKLQ)	6.2-12.5	1.6-3.1	12.5-25	39	50
**BPC076W**	c(KLKKKWLKLQ)	6.2-12.5	1.6-3.1	6.2-12.5	15	21
**BPC078W**	c(LKKKKWLKLQ)	6.2-12.5	0.8-1.6	25-50	3	6
**BPC080W**	c(KKKLLWKKLQ)	6.2-12.5	0.8-1.6	12.5-25	3	4
**BPC082W**	c(KKLKLWKKLQ)	6.2-12.5	1.6-3.1	12.5-25	26	34
**BPC084W**	c(KLKKLWKKLQ)	12.5-25	1.6-3.1	12.5-25	54	60
**BPC086W**	c(LKKKLWKKLQ)	3.1-6.2	0.8-1.6	6.2-12.5	4	8
**BPC088W**	c(KKLLKWKKLQ)	6.2-12.5	1.6-3.1	12.5-25	14	19
**BPC090W**	c(KLKLKWKKLQ)	6.2-12.5	1.6-3.1	12.5-25	9	15
**BPC092W**	c(LKKLKWKKLQ)	6.2-12.5	3.1-6.2	50-75	2	3
**BPC094W**	c(KLLKKWKKLQ)	6.2-12.5	3.1-6.2	25-50	35	42
**BPC096W**	c(LKLKKWKKLQ)	6.2-12.5	1.6-3.1	12.5-25	10	15
**BPC098W**	c(LLKKKWKKLQ)	3.1-6.2	1.6-3.1	12.5-25	12	18
**BPC100W**	c(KKKKLWLLKQ)	12.5-25	6.2-12.5	>100	19	27
**BPC102W**	c(KKKLKWLLKQ)	3.1-6.2	6.2-12.5	12.5-25	12	17
**BPC104W**	c(KKLKKWLLKQ)	6.2-12.5	6.2-12.5	12.5-25	2	3
**BPC106W**	c(KLKKKWLLKQ)	3.1-6.2	3.1-6.2	25-50	2	2
**BPC108W**	c(LKKKKWLLKQ)	1.6-3.1	1.6-3.1	6.2-12.5	4	5
**BPC110W**	c(KKKLLWKLKQ)	3.1-6.2	6.2-12.5	12.5-25	3	4
**BPC112W**	c(KKLKLWKLKQ)	3.1-6.2	6.2-12.5	50-75	3	4
**BPC114W**	c(KLKKLWKLKQ)	3.1-6.2	1.6-3.1	12.5-25	29	38
**BPC116W**	c(LKKKLWKLKQ)	3.1-6.2	3.1-6.2	12.5-25	9	14
**BPC118W**	c(KKLLKWKLKQ)	6.2-12.5	12.5-25	12.5-25	4	4
**BPC120W**	c(LKKLKWKLKQ)	3.1-6.2	1.6-3.1	12.5-25	6	9
**BPC122W**	c(KLLKKWKLKQ)	3.1-6.2	6.2-12.5	12.5-25	2	6
**BPC124W**	c(LKLKKWKLKQ)	6.2-12.5	3.1-6.2	50-75	2	2
**BPC126W**	c(LLKKKWKLKQ)	6.2-12.5	1.6-3.1	25-50	5	9
**BPC128W**	c(KKKLLWLKKQ)	6.2-12.5	1.6-3.1	12.5-25	2	2
**BPC130W**	c(KKLKLWLKKQ)	6.2-12.5	1.6-3.1	12.5-25	1	2
**BPC132W**	c(KLKKLWLKKQ)	3.1-6.2	1.6-3.1	12.5-25	5	8
**BPC134W**	c(LKKKLWLKKQ)	6.2-12.5	3.1-6.2	25-50	3	4
**BPC136W**	c(KKLLKWLKKQ)	6.2-12.5	3.1-6.2	6.2-12.5	1	2
**BPC138W**	c(KLKLKWLKKQ)	6.2-12.5	3.1-6.2	25-50	7	14

**BPC140W**	c(LKKLKWLKKQ)	6.2-12.5	3.1-6.2	12.5-25	31	37
**BPC142W**	c(KLLKKWLKKQ)	6.2-12.5	3.1-6.2	12.5-25	16	18
**BPC144W**	c(LKLKKWLKKQ)	3.1-6.2	1.6-3.1	12.5-25	16	21
**BPC146W**	c(LLKKKWLKKQ)	3.1-6.2	3.1-6.2	25-50	4	7
**BPC148W**	c(KKLLLWKKKQ)	12.5-25	12.5-25	>100	1	2
**BPC150W**	c(KLKLLWKKKQ)	12.5-25	6.2-12.5	>100	17	32
**BPC152W**	c(LKKLLWKKKQ)	6.2-12.5	6.2-12.5	75-100	1	3
**BPC154W**	c(KLLKLWKKKQ)	3.1-6.2	1.6-3.1	25-50	4	7
**BPC156W**	c(LKLKLWKKKQ)	6.2-12.5	3.1-6.2	>100	1	2
**BPC158W**	c(LLKKLWKKKQ)	12.5-25	1.6-3.1	12.5-25	10	14
**BPC160W**	c(KLLLKWKKKQ)	6.2-12.5	6.2-12.5	>100	3	6
**BPC162W**	c(LKLLKWKKKQ)	6.2-12.5	3.1-6.2	>100	57	56
**BPC164W**	c(LLKLKWKKKQ)	6.2-12.5	6.2-12.5	>100	6	6
**BPC166W**	c(LLLKKWKKKQ)	6.2-12.5	6.2-12.5	>100	1	1
**BPC184W**	c(KLLLKWKKLQ)	3.1-6.2	1.6-3.1	25-50	22	32
**BPC186W**	c(KKKLKWKKLQ)	12.5-25	6.2-12.5	>100	1	1
**BPC188W**	c(LLLKKWKKLQ)	6.2-12.5	12.5-25	50-75	33	48
**BPC190W**	c(LKKKKWKKLQ)	25-50	12.5-25	>100	1	3
**BPC192W**	c(LKLLKWKKLQ)	12.5-25	6.2-12.5	25-50	13	17
**BPC194W**	c(KKLKKWKKLQ)	12.5-25	6.2-12.5	12.5-25	3	5
**BPC196W**	c(LLKLKWKKLQ)	3.1-6.2	1.6-3.1	>100	2	3
**BPC198W**	c(KLKKKWKKLQ)	25-50	3.1-6.2	25-50	2	4
**BPC200W**	c(LLLLKWKKLQ)	>50	3.1-6.2	75-100	6	7
**BPC202W**	c(KKKKKWKKLQ)	25-50	12.5-25	>100	0	1

[a] Percent hemolysis at 50 and 125 μM.

[b] *Pss*, *Pseudomonas syringae* pv. *syringae*; *Xav*, *Xanthomonas axonopodis* pv. *vesicatoria*; *Ea*, *Erwinia amylovora*
